# Case Series of Laboratory-Associated Zika Virus Disease, United States, 2016–2019

**DOI:** 10.3201/eid2705.203602

**Published:** 2021-05

**Authors:** Susan L. Hills, Andrea Morrison, Shawna Stuck, Kayleigh Sandhu, Krystal L. Mason, Danielle Stanek, Julie Gabel, Matthew A. Osborne, Betsy A. Schroeder, Edhelene Rico, Cherie L. Drenzek, Glen R. Gallagher, Jennifer Fiddner, Lea A. Heberlein-Larson, Catherine M. Brown, Marc Fischer

**Affiliations:** Centers for Disease Control and Prevention, Fort Collins, Colorado, USA (S.L. Hills, M. Fischer);; Florida Department of Health, Tallahassee, Florida, USA (A. Morrison, D. Stanek);; Georgia Department of Public Health, Atlanta, Georgia, USA (S. Stuck, J. Gabel, C.L. Drenzek);; Massachusetts Department of Public Health, Jamaica Plain, Massachusetts, USA (K. Sandhu, M.A. Osborne, G.R. Gallagher, C.M. Brown);; Pennsylvania Department of Health, Harrisburg, Pennsylvania, USA (K.L. Mason, B.A. Schroeder);; Florida Department of Health in Miami-Dade County, Miami, Florida, USA (E. Rico);; Allegheny County Health Department, Pittsburgh, Pennsylvania, USA (J. Fiddner);; Florida Department of Health Bureau of Public Health Laboratories, Tampa, Florida, USA (L.A. Heberlein-Larson)

**Keywords:** Zika virus, laboratory, needlestick, occupational, viruses, zoonoses, vector-borne diseases, mosquito-borne diseases, flavivirus

## Abstract

Zika virus diagnostic testing and laboratory research increased considerably when Zika virus began spreading through the Americas in 2015, increasing the risk for potential Zika virus exposure of laboratory workers and biomedical researchers. We report 4 cases of laboratory-associated Zika virus disease in the United States during 2016–2019. Of these, 2 were associated with needlestick injuries; for the other 2 cases, the route of transmission was undetermined. In laboratories in which work with Zika virus is performed, good laboratory biosafety practices must be implemented and practiced to reduce the risk for infection among laboratory personnel.

Zika virus is a flavivirus that was first isolated in 1947 from a rhesus macaque in the Zika Forest in Uganda. Zika virus is primarily transmitted to humans by infected mosquitoes, but other confirmed transmission modes include intrauterine, sexual, and intrapartum transmission, and probable modes include transmission through blood transfusion and breastfeeding (*1*). Laboratory-associated infection also has been reported in a small number of cases; one of the earliest reports of human Zika virus infection was possibly laboratory-acquired (*2*). A researcher was working in a Uganda laboratory in 1963 with Zika virus strains isolated from mosquitoes. After he experienced fever and rash, laboratory testing indicated Zika virus infection. However, no apparent breakdown in biosafety procedures was identified, and mosquitoborne transmission could not be excluded. In 1972, Zika virus infection in another laboratory worker occurred, this time in the absence of a potential mosquitoborne route of transmission (*3*). The person was symptomatic, and infection was confirmed by virus isolation. He worked in an arboviral laboratory but no exposure that might have led to infection was reported. A 1980 report by the American Committee on Arthropod-borne Viruses, which documented results of global laboratory surveys conducted in 1976 and 1978, noted an additional 3 Zika virus disease cases in laboratory workers. The suspected sources of these infections were through the aerosol route or unknown, and further details were not provided (*4*). Finally, a laboratory-acquired Zika virus infection occurred in 2017 in Brazil after an infected mouse bit a researcher’s finger (*5*).

Zika virus diagnostic testing and laboratory research increased considerably beginning in 2015 when Zika virus began spreading through the Americas, increasing the risk for potential Zika virus exposure for laboratory workers and researchers. We report 4 cases of laboratory-associated Zika virus disease in the United States during 2016–2019.

## Case Reports

### Exposure to Zika Virus through Needlestick Injury

#### Case 1

In May 2016, a female researcher who worked in a Biosafety Level (BSL) 3 microbiology laboratory sustained a needlestick injury with a bifurcated needle; information on whether the skin was punctured was not available. The incident occurred during in vitro inoculation of human skin cells with wild-type Zika virus for vaccine research purposes. She was wearing 2 pairs of nitrile gloves and working in a biosafety cabinet. She immediately used a surgical sponge and chlorohexidine to scrub the wound for 15 minutes, then washed her hands with soap and water. After 9 days, she experienced a low-grade fever, generalized maculopapular rash, headache, myalgia, and fatigue; mild unilateral conjunctivitis occurred the next day. She did not live in an area with local Zika virus transmission, and in the month before illness onset she had no other risk factors for acquisition of Zika virus infection (i.e., no history of travel, no sexual contact with a traveler, and no history of blood transfusion or organ transplantation). She reported full resolution of her symptoms within 5 days. Zika virus infection was confirmed through the detection of Zika virus RNA in serum and urine and Zika virus IgM and neutralizing antibodies in serum (Table).

**Table:** Laboratory results from 4 patients with laboratory-associated Zika virus disease, United States, 2016–2019*

	Days post-onset of collection†	Sample type	Test conducted‡	Result
Case 1	1	Serum	RT-PCR	Zika virus RNA detected
	2	Serum	RT-PCR	Zika virus RNA detected
	2	Urine	RT-PCR	Zika virus RNA detected
	2	Serum	IgM ELISA	Zika virus IgM equivocal
	2	Serum	PRNT	Zika virus titer >20, DENV titer <10
Case 2	4	Serum	RT-PCR	Negative
	4	Urine	RT-PCR	Zika virus RNA detected
	4	Serum	IgM ELISA	Zika virus IgM positive
	4	Serum	PRNT	Zika virus titer <10, DENV titer <10
	20	Serum	RT-PCR	Negative
	20	Urine	RT-PCR	Negative
	20	Serum	IgM ELISA	Zika virus IgM positive
	20	Serum	PRNT	Zika virus titer 320, DENV titer 20
Case 3	2	Serum	RT-PCR	Zika virus RNA detected
	2	Serum	IgM ELISA	Negative
	20	Serum	RT-PCR	Negative
	20	Serum	IgM ELISA	Zika virus IgM positive
	20	Serum	PRNT	Zika virus titer >1280, DENV titer <10
	≈120	Semen	RT-PCR	Zika virus RNA detected
Case 4	5	Serum	RT-PCR	Negative
	5	Urine	RT-PCR	Zika virus RNA detected
	5	Serum	PRNT	Zika virus titer 160
	10	Urine	RT-PCR	Zika virus RNA detected
	10	Serum	RT-PCR	Negative
	10	Serum	IgM ELISA	Zika virus IgM positive
	10	Serum	PRNT	Zika virus titer 1280

*CDC, Centers for Disease Control and Prevention; DENV, dengue virus; PRNT, 90% plaque-reduction neutralization test; RT-PCR, reverse transcription PCR.  †Day 0 equals day of illness onset.  ‡Tests conducted at state public health laboratories, commercial laboratories, and CDC Arboviral Diseases Branch Reference Laboratory.

#### Case 2

In July 2018, a female researcher received an accidental needlestick injury while recapping a needle after inoculating a mouse with the Uganda Zika virus strain MR766 at a concentration of 10^7^ PFU/mL (*6*). At the time of the incident, she was working in a biosafety cabinet and was double gloved. She felt the stick from the needle on her left middle finger but did not see any blood. She immediately removed her gloves, washed her hands with soap and water, and applied alcohol. After 10 days, she became symptomatic with a pruritic maculopapular rash, arthralgia, and myalgia. Zika virus infection was confirmed on the basis of the detection of Zika virus RNA in urine and serologic testing (Table). There was no reported local Zika virus transmission where she lived, and apart from the needlestick injury she had no other risk factors for acquisition of Zika virus infection. She recovered completely within ≈2 weeks of symptom onset.

### Other Laboratory-Associated Zika Virus Exposures

#### Case 3

In November 2017, a male worker in a BSL-2 virology laboratory had onset of symptoms (day 0) of headache, arthralgia, myalgia, fatigue, and a rash that initially appeared on his face and spread to his whole body during the next 2 days. The arthralgia and myalgia became progressively more severe and debilitating through day 5, but recovery occurred by day 13. Zika virus infection was confirmed through detection of Zika virus RNA in serum and semen and with serologic methods (Table). He had no other risk factors for acquisition of infection and there was no local Zika virus transmission where he lived.

The patient reported that he typically worked with large quantities (4–100 L) of Zika virus in the laboratory but did not recall any specific exposure or incident of concern within the 2 weeks before illness onset. His activities included clarifying Zika virus materials through filters, performing pump-driven chromatography, using buffers to dilute concentrated Zika virus, and adding formaldehyde to initiate Zika virus inactivation. The recommended personal protective equipment (PPE) he routinely wore included a first PPE layer, donned in an external area, of disposable laboratory coat or coverall, booties, a hairnet, goggles, and 1 pair of gloves and a second PPE layer of a second coverall, hairnet, pair of gloves, and disposable face shield donned once inside the laboratory; no mask was used. He performed his work inside a biosafety cabinet when possible but could not do so when using larger containers (e.g., the biosafety cabinet could not accommodate the large vessels used for pouring liquid live virus through a funnel). The liquid could sometimes potentially splash. On 1 occasion during the probable exposure period, while he was working in a biosafety cabinet, a large droplet of live virus dripped onto his glove; he immediately changed the outer glove. He reported it was possible he might have rubbed his face with the back of a gloved hand; however, no confirmed mucus membrane exposure could be identified. An additional 12 employees working with Zika virus in the same laboratory were subsequently tested and showed no serologic evidence of recent or past Zika virus infection.

#### Case 4

In October 2019, a male researcher in a vaccine research laboratory experienced fever, rash, arthralgia, and conjunctival injection. His laboratory activities sometimes involved working with Zika virus, including performing serum neutralization testing, and he had worked with Zika virus 8 and 10 days before symptom onset. He routinely wore gloves in the laboratory, but more detailed PPE information was unavailable. An investigation did not identify any specific exposure or reported breach in biosafety procedures, and no sharps were used in the laboratory. He did not live in an area with a history of Zika virus transmission and he had no other risk factors for Zika virus infection. Confirmation of infection was by detection of Zika virus RNA in urine and by serologic methods (Table). Symptoms resolved within 8 days.

## Discussion

During the 4-year period from 2016–2019, 4 cases of laboratory-acquired Zika virus infection were reported in the United States: 2 associated with needlestick injuries and 2 in which the means of exposure was undetermined. In laboratories where work with Zika virus is performed, good laboratory safety practices are critical to reduce the risk to personnel of Zika virus exposure and disease.

Many factors affect the likelihood of Zika virus infection following exposure, including the type and severity of any injury or exposure, route of exposure, viral concentration and dose, transmissibility of the strain, immediate management of any recognized exposure, and the worker’s health status. At least 3 other potential occupational exposures to Zika virus have occurred among researchers without subsequent Zika virus infection: a bite from an infected mouse that punctured the skin of a gloved researcher’s finger (*7*), a puncture wound from a needle that occurred when a double-gloved researcher was collecting a blood sample from a Zika virus-infected ferret (M. Sauri, Occupational Health Consultants, pers. comm., 2017 Jan 30), and a thumb laceration from a scalpel contaminated with chicken blood in a researcher harvesting chickens inoculated with Zika virus (*7*). Other exposures or infections might have occurred and remained unreported or been undetected if appropriate testing was not completed.

A limitation of this report is that viral sequencing could not be done to provide supporting evidence that the Zika virus infections were laboratory-acquired. However, the patients lived in areas without endemic Zika virus disease and patient investigations revealed no other risk factors for acquisition of Zika virus infection (i.e., no patients had traveled, had sexual contact with a traveler, or received a blood transfusion or organ transplant). Therefore, the infections were likely laboratory-acquired.

The Biosafety in Microbiological and Biomedical Laboratories guidelines recommend BSL-2 practices, safety equipment, and facilities for working with Zika virus (*8*). Similarly, recommendations exist for animal BSL-2 practices, equipment, and facility requirements when animal studies involving Zika virus are conducted (*8*). In addition, laboratories should perform a risk assessment to determine whether certain procedures or specimens might require higher levels of biocontainment (*9*). For example, manipulating large quantities of virus or high titer preparations might warrant a shift to BSL-3 practices, including additional respiratory protection (*8*). Altering practices might be particularly critical when working outside a biosafety cabinet or when not wearing adequate PPE to protect against aerosol or droplet transfer of infectious material.

Laboratory personnel should have appropriate training regarding precautions to prevent exposures associated with the tasks they perform (*8*). Institutional policies also should be in place and accessible. Because careful management of needles and other sharps is vital, policies should include recommendations for the safe handling of sharps; for needles, actions that involve manipulation by hand before disposal, including bending, recapping, or removing from the syringe, are not advised (*8*). Biosafety in Microbiological and Biomedical Laboratories guidelines provide comprehensive information on recommended practices, safety equipment, and laboratory facilities (*8*). Broader guidance for protecting workers from occupational exposure to Zika virus also is available from the Occupational Safety and Health Administration and from the Centers for Disease Control and Prevention National Institute for Occupational Safety and Health (*10*).

Appropriate evaluation and management of occupational Zika virus exposures is crucial. If an incident occurs, established workplace procedures for initial wound management or mucous membrane exposures should be followed and the event immediately reported to a supervisor. No specific Zika virus post-exposure prophylaxis exists; however, as soon as possible after the incident, a baseline serum sample should be obtained and stored in case comparison with a convalescent serum sample is needed. Persons should be advised to take steps to prevent potential sexual transmission of Zika virus and to avoid mosquito bites if in a geographic area with risk for mosquito-borne transmission of Zika virus. These measures should be continued until laboratory testing excludes infection; if Zika virus infection is confirmed, additional counseling should be provided. If symptoms consistent with Zika virus disease occur within 2 weeks of the exposure, serum and urine should be collected and tested by using appropriate molecular and serologic methods. For an exposed person who remains asymptomatic, a serum sample should be obtained >2 weeks postexposure. This serum sample should be tested for Zika virus IgM and if positive, tested by plaque-reduction neutralization test, and results compared with those from the baseline sample to assess for asymptomatic infection. Similarly, if a person is symptomatic within 2 weeks of exposure and test results on collected samples are negative, indicating the illness is unrelated to Zika virus infection, consideration should be given to obtaining an additional serum sample at >2 weeks postexposure and similarly evaluating for asymptomatic infection.

Although Zika virus transmission has declined substantially in recent years, research using Zika virus is ongoing. Exposure and infection are occupational risks for laboratory and biomedical research workers who work with live virus. Strong infection prevention practices are essential for reducing this risk (*11*). Establishing and implementing appropriate policies and procedures, providing adequate training, making available and ensuring proper use of PPE and other safety equipment, and confirming facilities are suitable for the type of work being conducted are all required to protect personnel from any adverse health outcomes.

## References

[1] Gregory CJ, Oduyebo T, Brault AC, Brooks JT, Chung KW, Hills S, et al. Modes of transmission of Zika virus. J Infect Dis. 2017;216(suppl_10):S875–83. 10.1093/infdis/jix396 29267909 PMC5853257

[2] Simpson DI. Zika virus infection in man. Trans R Soc Trop Med Hyg. 1964;58:335–8. 10.1016/0035-9203(64)90201-9 14175744

[3] Filipe AR, Martins CMV, Rocha H. Laboratory infection with Zika virus after vaccination against yellow fever. Arch Gesamte Virusforsch. 1973;43:315–9. 10.1007/BF01556147 4799154

[4] The Subcommittee on Arbovirus Laboratory Safety of the American Committee on Arthropod-Borne Viruses. Laboratory safety for arboviruses and certain other viruses of vertebrates. Am J Trop Med Hyg. 1980;29:1359–81. 10.4269/ajtmh.1980.29.1359 6778230

[5] Talon de Menezes M, Rilo Christoff R, Higa LM, Pezzuto P, Rabello Moreira FR, Ribeiro LJ, et al. Laboratory acquired Zika virus infection through mouse bite: a case report. Open Forum Infect Dis. 2020;7:ofaa259. 10.1093/ofid/ofaa259 PMC7686659 33269292

[6] Lichtenberger PN, Ricciardi MJ, Solorzano D, Raccamarich P, Leda A, Sharkey M, et al. Occupational exposure to the Ugandan research strain (MR766) of Zika virus. Open Forum Infect Dis. 2019;6:ofz420. 10.1093/ofid/ofz420 31667199 PMC6814281

[7] Shugart JM, Brown CK. Zika virus presents an ongoing occupational health hazard for laboratory and biomedical research workers. J ABSA Int. 2019;24:8–9. 10.1177/1535676018818562 PMC9093242 36034635

[8] US Department of Health and Human Services. Biosafety in microbiological and biomedical laboratories. 5th edition. 2009 [cited 2020 Feb 5]. https://www.cdc.gov/labs/pdf/CDC-BiosafetyMicrobiologicalBiomedicalLaboratories-2009-P.PDF

[9] Centers for Disease Control and Prevention. Laboratory safety when working with Zika virus [cited 2020 Feb 5]. https://www.cdc.gov/zika/laboratories/lab-safety.html

[10] Occupational Safety and Health Administration, National Institute for Occupational Safety and Health. Interim guidance for protecting workers from occupational exposure to Zika virus [cited 2020 Feb 5]. https://www.osha.gov/zika

[11] Brown CK, Shugart JM. Zika virus in workers: Considerations for ongoing exposure prevention. Am J Ind Med. 2019;62:455–9. 10.1002/ajim.22978 31025402

